# Effect of nano-hydroxyapatite toothpaste on microhardness ofartificial carious lesions created on extracted teeth

**DOI:** 10.15171/joddd.2017.003

**Published:** 2017-03-15

**Authors:** Asghar Ebadifar, Mohammad Nomani, Sayed Ali Fatemi

**Affiliations:** ^1^Dentofacial Deformities Research Center, Research Institute of Dental Sciences & Department of Orthodontics, Dental School, Shahid Behehsti University of Medical Sciences, Tehran, Iran; ^2^Dentist, Private Practice, Tehran, Iran; ^3^Department of Pharmacology and Toxicology, Faculty of Pharmacy, Isfahan University of Medical Sciences, Isfahan, Iran

**Keywords:** Toothpaste, nano-hydroxyapatite, microhardness, remineralization, decalcification

## Abstract

***Background.*** Many types of toothpastes contain substances that can remineralize initial enamel caries. This study aimed to assess the effect of nano-hydroxyapatite (NHA) on microhardness of artificially created carious lesions.

***Methods.*** In this in vitro study, NHA was prepared using sol-gel technique and added to the toothpaste with 7% concentration. A total of 80 extracted sound teeth were collected. The crowns were polished using 500-grit abrasive paper. The specimens were randomly coded from 1 to 80. Number 1 to 40 were assigned to group A and numbers 41 to 80 to group B. The microhardness was measured using HVS-1000 Vickers microhardness tester. The specimens were demineralized using 37% phosphoric acid for 3 minutes in order to create artificial carious lesions and then were rinsed with water, air-sprayed for 3 minutes and dried. Microhardness was measured again. Next, the specimens were brushed for 15 days, twice daily, for 15 seconds. After 15 days, microhardness was measured again. Toothpaste A contained NHA and fluoride and toothpaste B contained fluoride alone. Data were analyzed using SPSS 16, with one-sample Kolmogorov-Smirnov test and ANOVA at a significance level of P<0.05.

***Results.*** The microhardness of specimens significantly decreased following acid exposure (P<0.01) but increased again in both groups after exposure to toothpastes. The increase in microhardness was significantly greater in group A (P<0.01).

***Conclusion.*** The toothpaste containing NHA was more effective than the toothpaste without NHA for the purpose of remineralization.

## Introduction

Dental caries is a complex multifactorial disease that affects the majority of the population regardless of gender, ethnicity and age.^1^ Although preventable, dental caries still remains a major concern in developing countries and is known as the most common chronic childhood disease with a prevalence rate 5 and 7 times that of asthma and hay fever, respectively.^2^ Initial caries progression may be prevented by suitable surface treatment. This issue signifies current concept regarding remineralization and demineralization of the tooth surface.^2^ For about 100 years, tooth surface remineralization methods have been studied. Remineralizing agents are produced in various forms such as restorative materials, fissure sealants, chewing gums, mouth rinses and dentifrices.^3,4^ One of the most effective remineralizing agents in caries prevention is fluoride. Nevertheless, some concerns have been expressed about fluorosis and total fluoride intake.^3,4^ In recent years, fluoride alternatives have been proposed, including CPP-ACP and nano-hydroxyapatite (NHA) because of their anticariogenic characteristics.^5,6^ HA contains calcium and phosphate crystals that are found in cementum, enamel and dentin. Because of similar characteristics to human hard tissue, biocompatibility and low solubility in humid environments, HA has received great attention in medicine, dentistry and biology. Antibacterial effect of HA is one of the most important properties that has been shown in several studies.^7^ Many studies have used HA for enamel lesion repair due to its chemical and structural similarity to tooth mineral content.^8^ NHA toothpastes were first investigated in Japan in the 1980s. Studies have reported more or comparable remineralizing effects for NHA toothpastes in comparison to other toothpastes containing aminofluoride and fluoride.^9,10^ Daily tooth brushing with NHA toothpaste can provide adequate amounts of HA and enrich the saliva and dental plaque to prevent the progression of initial caries.^11^ The aim of the present study was to assess the effect of toothpastes containing NHA in comparison to fluoridated toothpaste on microhardness of initial dentin carious lesions.

## Methods

This in vitro study was conducted on 80 extracted premolars that were free from caries, cracks, wear and hypocalcification on clinical examination. The tooth surfaces were then mechanically cleaned from debris and calculus using fluoride-free, prophylactic pumice paste, rubber cups and a low-speed handpiece (W&H, Bürmoos, Austria). The teeth were then evaluated under a stereomicroscope‏) Carton Industries Ltd, SCW, E, Thailand) at ×40 magnification for any enamel defects, carious lesions or cracks. The roots were cut using a cutting saw (Proxxon 37160 KGS 80 MICRO Saw Germany Föhren) and the teeth were then embedded and fixed in auto-polymerizing polymethyl methacrylate acrylic resin in specific blocks prepared for this purpose. The specimens were then randomly coded from 1 to 80. The surface of specimens (root dentin) was polished. For accurate measurement of microhardness, the surface of specimens was polished with 500-girt abrasive paper under water irrigation to achieve a smooth surface for evaluation under Vickers microhardness tester. After polishing, the specimen surfaces were dried and the baseline microhardness of specimens was measured using Vickers microhardness tester (Shimadzu M g5037, Japan). The best point for load application was determined and a 200-g load was applied for 10 seconds to three points on the specimen surface. After measuring the primary microhardness, each tooth was exposed to 37% phosphoric acid (Kimia, Tehran, Iran) for 3 minutes. After exposure, the teeth were rinsed using water-and-air spray and the secondary microhardness was measured using Vickers microhardness tester. The specimens, randomly coded from 1 to 80, were divided into two groups. Teeth #1 to #40 were brushed with toothpaste A and teeth #41 to #80 were brushed with toothpaste B.

Group A: Teeth in this group were brushed with toothpaste A (Goltash, Isfahn, Iran), containing 7% NHA and fluoride (NaF1000 PPM).

Group B: Teeth in this group were brushed with toothpaste B (Goltash, Isfahn, Iran), containing fluoride only (NaF, 1000 PPM).

All the specimens were brushed with a soft toothbrush and the respective toothpaste for 30 cycles 15 seconds each. The specimens were brushed twice daily and stored in tap water between the brushing cycles. During tooth brushing, the teeth were immersed in slurry water made of the respective toothpaste. The brushing intervention lasted for 15 days and after that, the specimens were dried and their microhardness was measured again. Data were analyzed using SPSS 16, with one-sample Kolmogorov-Smirnov test and ANOVA at a significance level of P<0.05. The statistician and the operator were both blinded to the group allocation of specimens.

## Results

Based on Kolmogorov-Smirnov test, data were distributed normally in all the groups at all the time intervals with minimum probability of 0.345. Thus, for the comparison of the two groups after controlling for the effect of hardness variables before and after the intervention (Table 1), ANOVA was applied.

**Table 1:** The means and standard deviations of microhardness values in the two groups at different time intervals

**Group**	**N**	**Mean ± SD**
**Before acid exposure**		
NHA toothpaste	40	70.3 ± 3.4
F toothpaste	40	63.6 ± 3.1
**After acid exposure**		
NHA toothpaste	40	32.9± 1.0
F toothpaste	40	36.2± 1.0
**After the intervention**		
**NHA toothpaste**	40	46.9 ± 1.3
F toothpaste	40	42.4 ± 1.7

After adjusting for the existing differences in terms of hardness before and after acid exposure using ANOVA, it was found that the two groups had significant differences in terms of final hardness (P<0.01). The hardness of specimens significantly decreased to 45% of the baseline value in group A and 57% of the baseline value in group B (P<0.01) after acid exposure and increased again after 15 days of exposure to toothpastes in both groups. The increase in microhardness was greater in the group A (NHA+F) than that in group B (F) (P<0.01).

## Discussion

Tooth decay is a pathological process characterized by the local destruction of teeth by cariogenic microorganisms following fermentation of carbohydrates and organic acids, resulting in tooth demineralization. It remains a public health dilemma in most communities.^12^ Because of infectious and contagious nature of decay, it is necessary to prevent it via controlling the contributing factors. Use of NHA toothpaste is a new technique for prevention of caries. NHA-containing toothpastes are now commercially produced by many manufacturers worldwide. Increased use of these toothpastes indicates the need for more precise evaluation of their efficacy. The aim of the present study was to evaluate the effect of NHA-containing toothpastes in comparison to fluoridated toothpastes on microhardness of initial carious lesions. The results showed significantly higher dentin microhardness following application of both of the toothpastes. NHA- containing toothpaste exhibited a higher remineralizing effect than fluoridated toothpaste (143% vs 116%). Yuan et al^14^ (2012) used 48 dentin specimens to compare the efficacy of 3% NHA and conventional toothpastes. Based on energy-dispersive spectrometer (EDS), they concluded that NHA was highly capable of obstructing the tubules and remineralizing the tooth structure, which is in accord with our study results.

Tschoppe et al^10^ (2011) prepared 85 dentin blocks of bovine teeth to compare the efficacy of 7%, 20% and 24% NHA toothpastes and 0.14% amine fluoride. By using microradiography, they found that NHA toothpastes had greater efficacy for remineralization of initial lesions compared to amine fluoride.

Huang et al^15^ (2009) prepared 129 bovine enamel blocks to compare the efficacy of 1%, 5%, 10% and 15% NHA and sodium fluoride. Vickers microhardness tester and scanning electron microscopy were used. They found that optimal concentration (10%) of NHA resulted in remineralization of initial enamel lesions.

HA, which is a bioactive and biocompatible material, is one of the primary components of tooth mineral content. HA is expected to significantly enhance remineralization of initial enamel and dentin caries, and NHA is believed to have a higher efficacy than HA for this purpose due to its nano-size particles (11). NHA has hydrophilic and wetting characteristics and is capable of producing a thin but tightly bound layer on the tooth surface, resulting in higher surface hardness and remineralization. HA is capable of obstructing the dentinal tubules and thus, relieves tooth hypersensitivity.^13^

Najibfard et al^11^ (2011) compared 10% and 5% NHA and a combination of 10% NHA + 1100 ppm NAF in an in vivo study. Based on microradiographs, the results showed comparable remineralizing effect of the toothpastes evaluated. This finding is in contrast to our findings, which might be attributed to differences in concentration of NHA and study designs. We used Vickers microhardness tester but Najibfard et al evaluated the remineralizing effect with microradiography.

King et al^15^ (2006) prepared enamel blocks of extracted human third molars in an in vitro study to compare the efficacy of 10% NHA toothpaste, 900 ppm sodium fluoride and 900 ppm sodium monofluorophosphate (MPF). The results showed that all the three agents remineralized enamel lesions and no significant difference was noted between their efficacy. Differences in the results might be explained by the use of polarized light microscopy and microradiography and the differences in the materials used.

Haghgoo et al^16^ (2014) found no differences between NHA and NaF mouthwashes in remineralizing effect. However, surface microhardnes and tooth remineralization significantly increased. They used the remineralizing agent in the form of a mouthwash.

Our results showed that a higher remineralizing effect of NHA toothpaste compared to NaF toothpaste. However, when using Vickers microhardness tester for the assessment of remineralizing effect of agents, the researchers should be well aware of the limitations of this method and generalization of in vitro results to the clinical setting. This method cannot completely simulate the oral conditions. Furthermore, this study evaluated the efficacy of a domestically made toothpaste containing NHA, which is more affordable than similar foreign products.

## Conclusion

Although fluoridated toothpaste can remineralize initial carious lesions, the synergistic effect of NHA (7 w%) and fluoride was shown.

## Acknowledgements

The authors would like to thank Goltash Co. and Dentofacial Deformities Research Center for supporting this study.

## Funding

This study was financially supported by both Goltash Co. and Dentofacial Deformities Research Center at Shahid Behehsti University of Medical Sciences. Neither of the funding bodies did not have a role in the conducting, analysis, nor reporting of this paper.

## Competing interests

The authors declare no competing interests with regards to the authorship and/or publication of this article.

## Ethics approval

The study protocol was approved by the Research Ethics Committee of Shahid Behehsti University of Medical Sciences.
